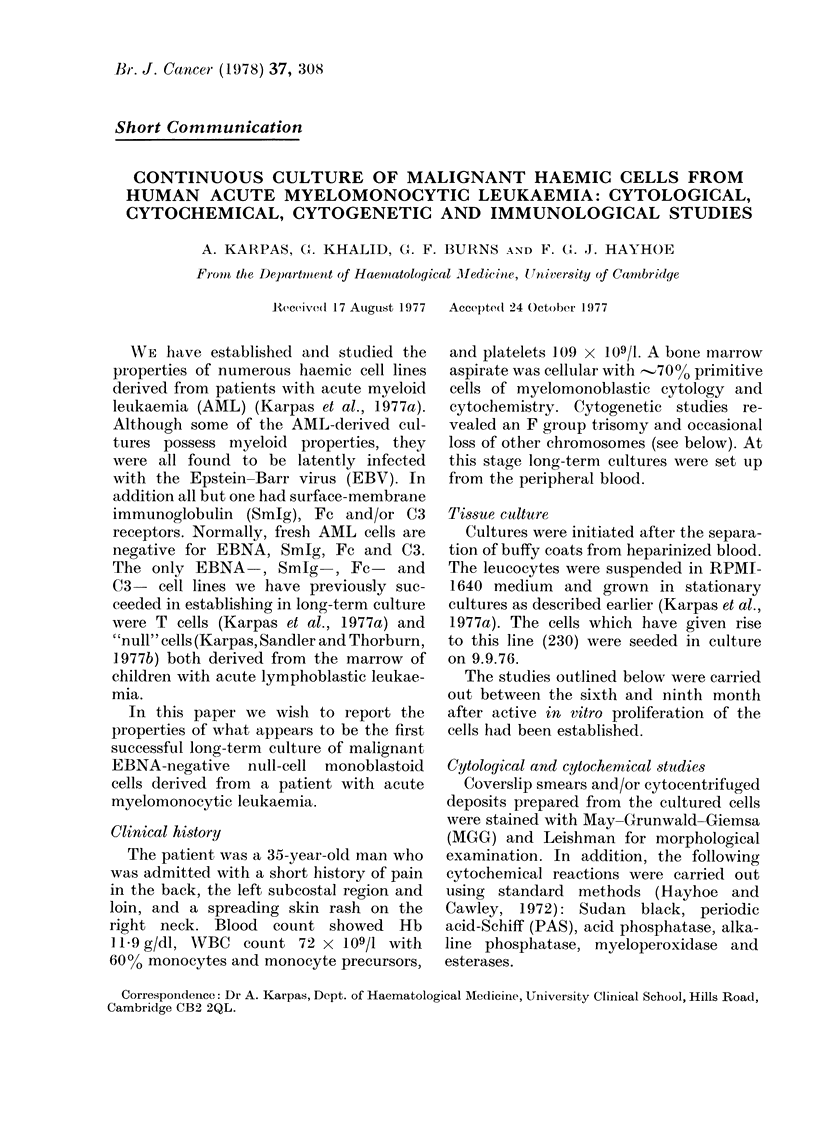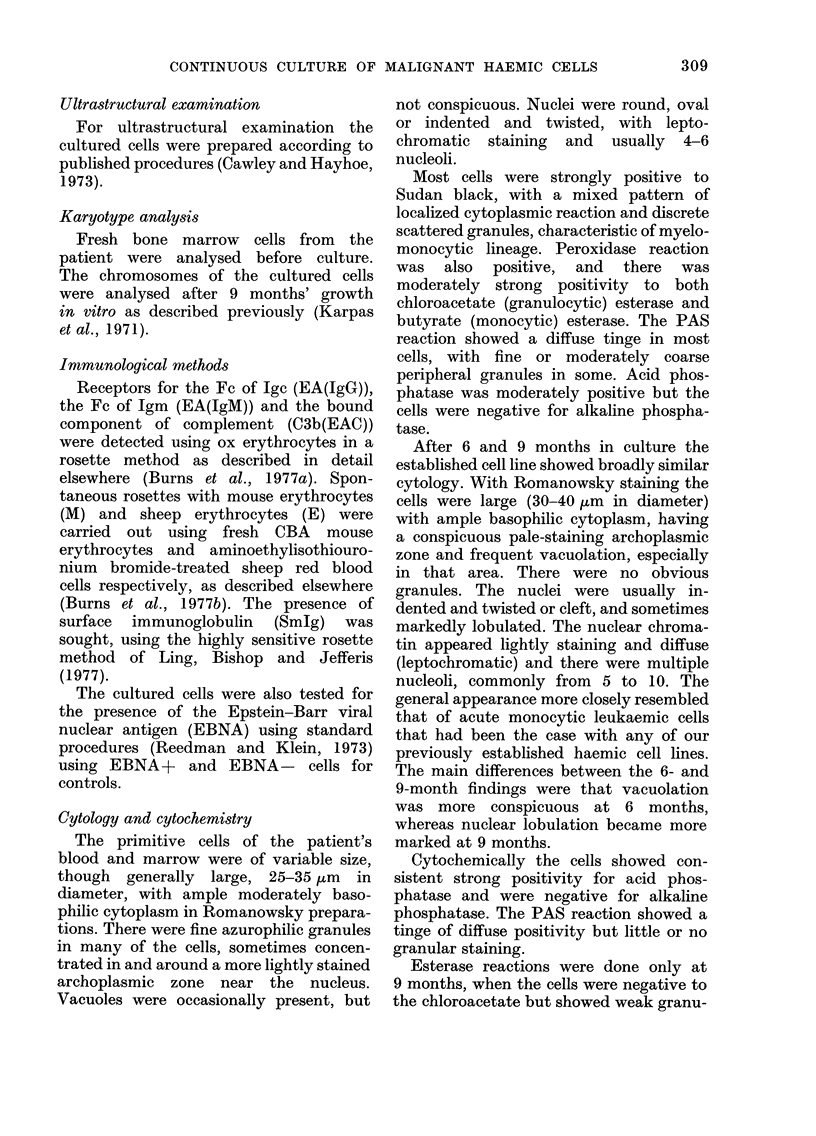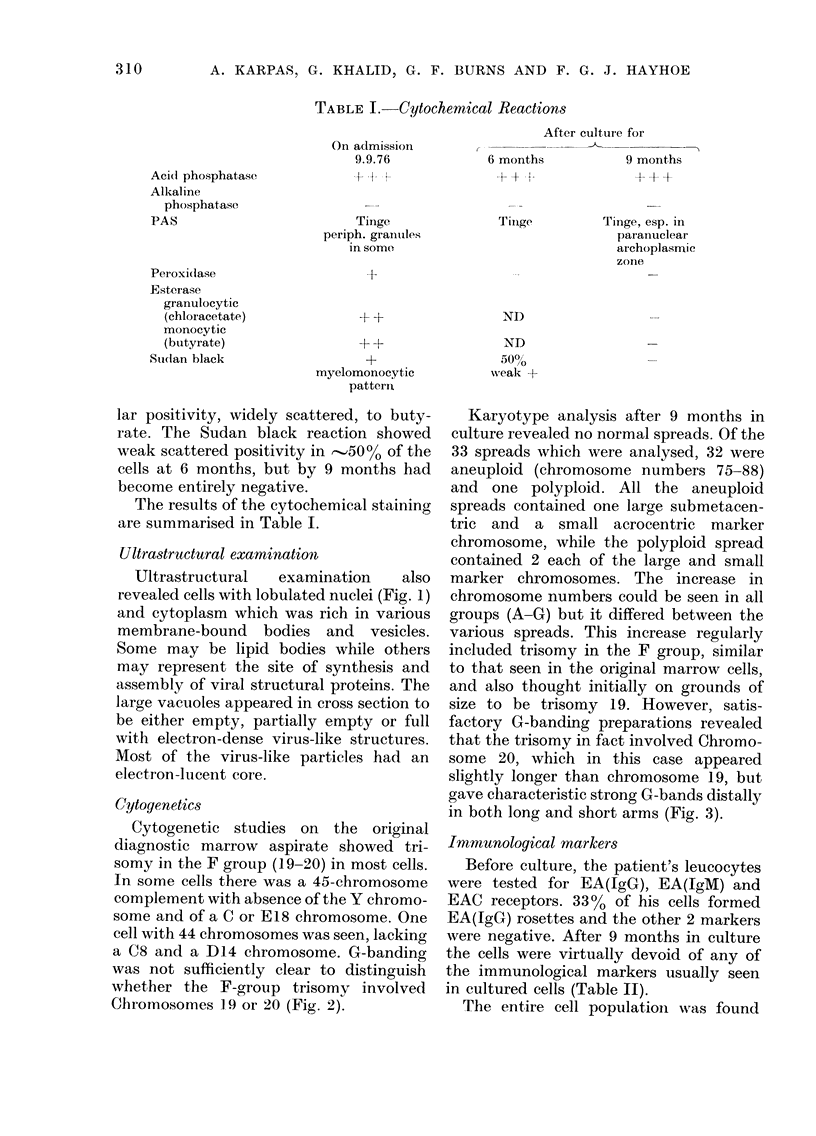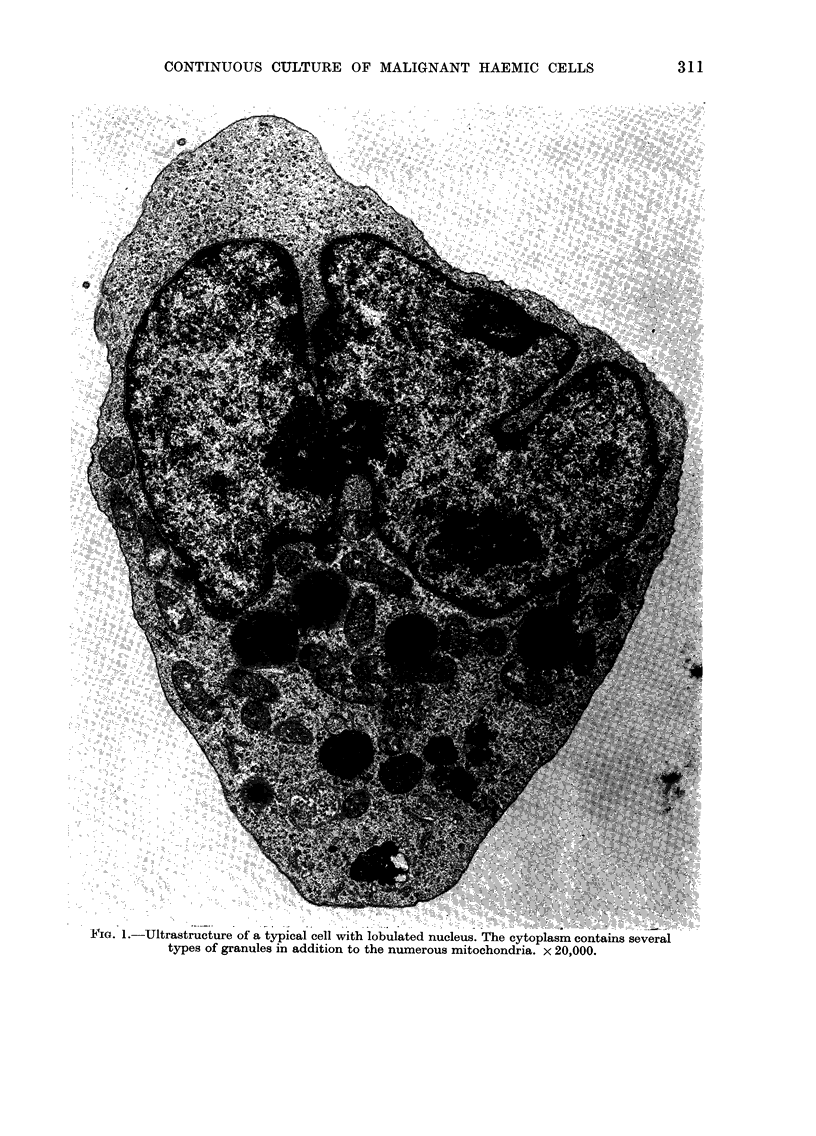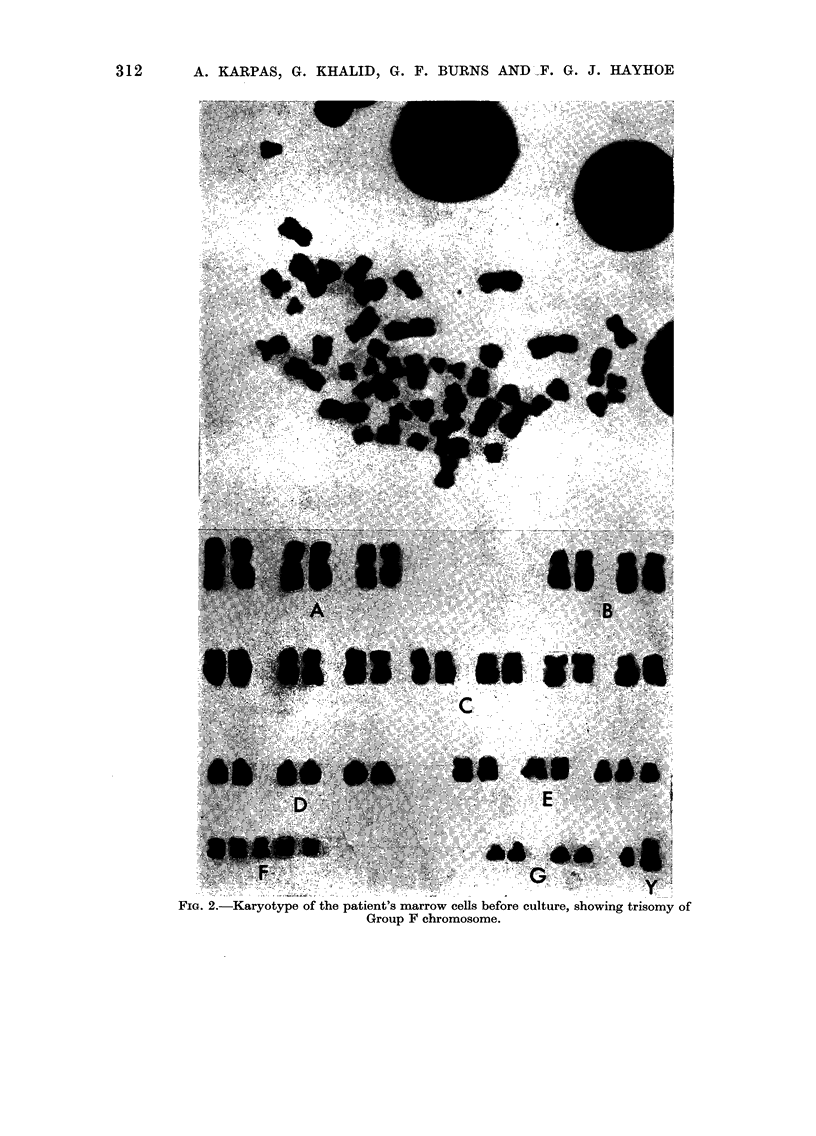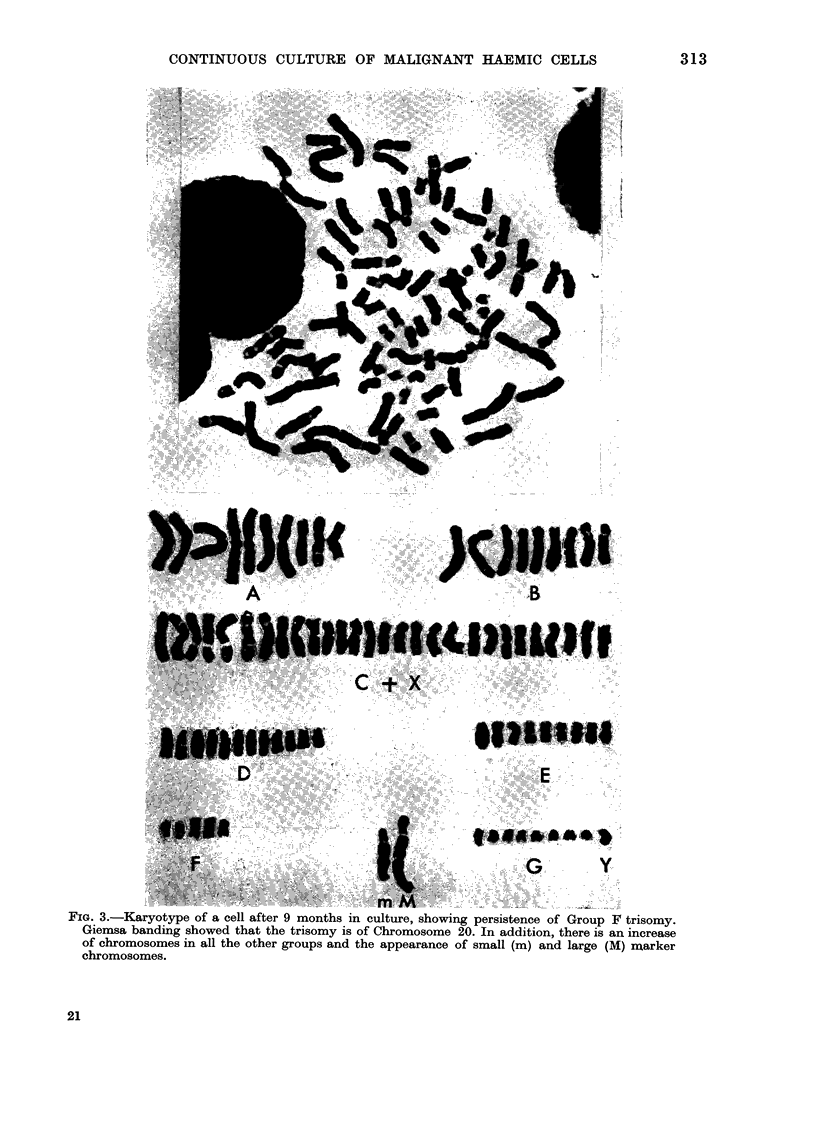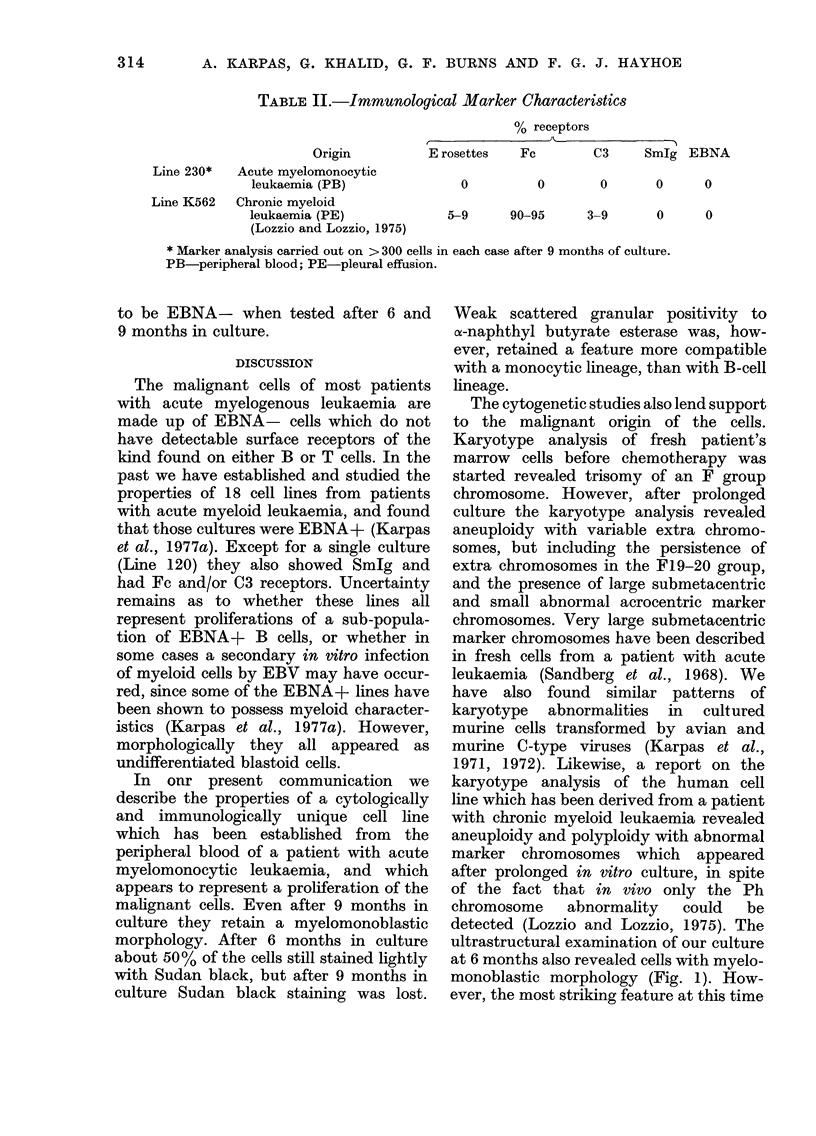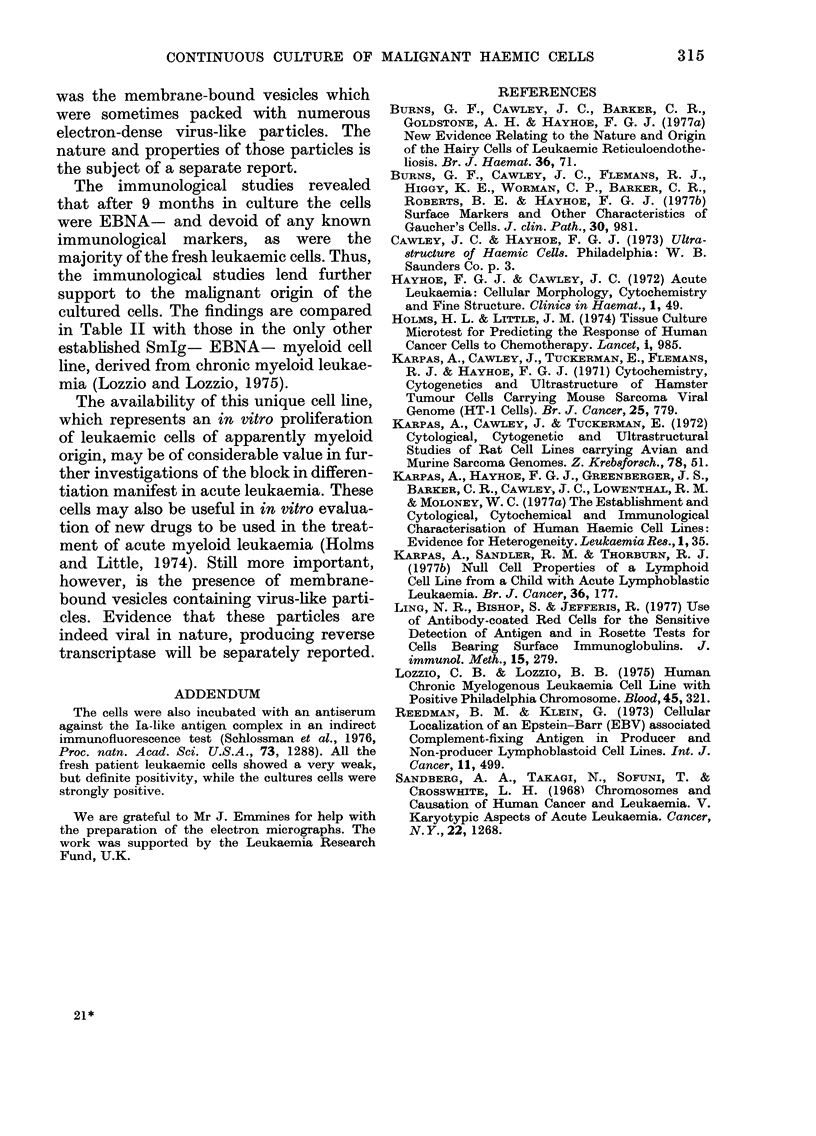# Continuous culture of malignant haemic cells from human acute myelomonocytic leukaemia: cytological, cytochemical, cytogenetic and immunological studies.

**DOI:** 10.1038/bjc.1978.40

**Published:** 1978-02

**Authors:** A. Karpas, G. Khalid, G. F. Burns, F. G. Hayhoe

## Abstract

**Images:**


*Br. J. Cancer* (1978) **37**, 308

***Short Communication***

### CONTINUOUS CULTURE OF MALIGNANT HAEMIC CELLS FROM HUMAN ACUTE MYELOMONOCYTIC LEUKAEMIA: CYTOLOGICAL, CYTOCHEMICAL, CYTOGENETIC AND IMMUNOLOGICAL STUDIES

A. KARPAS, G. KHALID, G. F. BURNS AND F. G. J. HAYHOE

*From the Department of Haematological Medicine, University of Cambridge*

Received 17 August 1977 Accepted 24 October 1977

WE have established and studied the properties of numerous haemic cell lines derived from patients with acute myeloid leukaemia (AML) (Karpas *et al.*, 1977*a*). Although some of the AML-derived cultures possess myeloid properties, they were all found to be latently infected with the Epstein–Barr virus (EBV). In addition all but one had surface-membrane immunoglobulin (SmIg), Fc and/or C3 receptors. Normally, fresh AML cells are negative for EBNA, SmIg, Fc and C3. The only EBNA−, SmIg−, Fc− and C3− cell lines we have previously succeeded in establishing in long-term culture were T cells (Karpas *et al.*, 1977*a*) and "null" cells (Karpas, Sandler and Thorburn, 1977*b*) both derived from the marrow of children with acute lymphoblastic leukaemia.

In this paper we wish to report the properties of what appears to be the first successful long-term culture of malignant EBNA-negative null-cell monoblastoid cells derived from a patient with acute myelomonocytic leukaemia.

*Clinical history*

The patient was a 35-year-old man who was admitted with a short history of pain in the back, the left subcostal region and loin, and a spreading skin rash on the right neck. Blood count showed Hb 11·9 g/dl, WBC count 72 × $10^9$/l with 60% monocytes and monocyte precursors, and platelets 109 × $10^9$/l. A bone marrow aspirate was cellular with ~70% primitive cells of myelomonoblastic cytology and cytochemistry. Cytogenetic studies revealed an F group trisomy and occasional loss of other chromosomes (see below). At this stage long-term cultures were set up from the peripheral blood.

*Tissue culture*

Cultures were initiated after the separation of buffy coats from heparinized blood. The leucocytes were suspended in RPMI-1640 medium and grown in stationary cultures as described earlier (Karpas *et al.*, 1977*a*). The cells which have given rise to this line (230) were seeded in culture on 9.9.76.

The studies outlined below were carried out between the sixth and ninth month after active *in vitro* proliferation of the cells had been established.

*Cytological and cytochemical studies*

Coverslip smears and/or cytocentrifuged deposits prepared from the cultured cells were stained with May–Grunwald–Giemsa (MGG) and Leishman for morphological examination. In addition, the following cytochemical reactions were carried out using standard methods (Hayhoe and Cawley, 1972): Sudan black, periodic acid-Schiff (PAS), acid phosphatase, alkaline phosphatase, myeloperoxidase and esterases.

Correspondence: Dr A. Karpas, Dept. of Haematological Medicine, University Clinical School, Hills Road, Cambridge CB2 2QL.

*Ultrastructural examination*

For ultrastructural examination the cultured cells were prepared according to published procedures (Cawley and Hayhoe, 1973).

*Karyotype analysis*

Fresh bone marrow cells from the patient were analysed before culture. The chromosomes of the cultured cells were analysed after 9 months' growth *in vitro* as described previously (Karpas *et al.*, 1971).

*Immunological methods*

Receptors for the Fc of Igc (EA(IgG)), the Fc of Igm (EA(IgM)) and the bound component of complement (C3b(EAC)) were detected using ox erythrocytes in a rosette method as described in detail elsewhere (Burns *et al.*, 1977*a*). Spontaneous rosettes with mouse erythrocytes (M) and sheep erythrocytes (E) were carried out using fresh CBA mouse erythrocytes and aminoethylisothiouronium bromide-treated sheep red blood cells respectively, as described elsewhere (Burns *et al.*, 1977*b*). The presence of surface immunoglobulin (SmIg) was sought, using the highly sensitive rosette method of Ling, Bishop and Jefferis (1977).

The cultured cells were also tested for the presence of the Epstein–Barr viral nuclear antigen (EBNA) using standard procedures (Reedman and Klein, 1973) using EBNA+ and EBNA− cells for controls.

*Cytology and cytochemistry*

The primitive cells of the patient's blood and marrow were of variable size, though generally large, 25–35 μm in diameter, with ample moderately basophilic cytoplasm in Romanowsky preparations. There were fine azurophilic granules in many of the cells, sometimes concentrated in and around a more lightly stained archoplasmic zone near the nucleus. Vacuoles were occasionally present, but not conspicuous. Nuclei were round, oval or indented and twisted, with leptochromatic staining and usually 4–6 nucleoli.

Most cells were strongly positive to Sudan black, with a mixed pattern of localized cytoplasmic reaction and discrete scattered granules, characteristic of myelomonocytic lineage. Peroxidase reaction was also positive, and there was moderately strong positivity to both chloroacetate (granulocytic) esterase and butyrate (monocytic) esterase. The PAS reaction showed a diffuse tinge in most cells, with fine or moderately coarse peripheral granules in some. Acid phosphatase was moderately positive but the cells were negative for alkaline phosphatase.

After 6 and 9 months in culture the established cell line showed broadly similar cytology. With Romanowsky staining the cells were large (30–40 μm in diameter) with ample basophilic cytoplasm, having a conspicuous pale-staining archoplasmic zone and frequent vacuolation, especially in that area. There were no obvious granules. The nuclei were usually indented and twisted or cleft, and sometimes markedly lobulated. The nuclear chromatin appeared lightly staining and diffuse (leptochromatic) and there were multiple nucleoli, commonly from 5 to 10. The general appearance more closely resembled that of acute monocytic leukaemic cells that had been the case with any of our previously established haemic cell lines. The main differences between the 6- and 9-month findings were that vacuolation was more conspicuous at 6 months, whereas nuclear lobulation became more marked at 9 months.

Cytochemically the cells showed consistent strong positivity for acid phosphatase and were negative for alkaline phosphatase. The PAS reaction showed a tinge of diffuse positivity but little or no granular staining.

Esterase reactions were done only at 9 months, when the cells were negative to the chloroacetate but showed weak granu-

TABLE I.—*Cytochemical Reactions*

| | On admission 9.9.76 | After culture for 6 months | After culture for 9 months |
|---|---|---|---|
| Acid phosphatase | +++ | +++ | +++ |
| Alkaline phosphatase | — | — | — |
| PAS | Tinge periph. granules in some | Tinge | Tinge, esp. in paranuclear archoplasmic zone |
| Peroxidase | + | — | — |
| Esterase granulocytic (chloracetate) | ++ | ND | — |
| monocytic (butyrate) | ++ | ND | — |
| Sudan black | + myelomonocytic pattern | 50% weak + | — |

lar positivity, widely scattered, to butyrate. The Sudan black reaction showed weak scattered positivity in ~50% of the cells at 6 months, but by 9 months had become entirely negative.

The results of the cytochemical staining are summarised in Table I.

*Ultrastructural examination*

Ultrastructural examination also revealed cells with lobulated nuclei (Fig. 1) and cytoplasm which was rich in various membrane-bound bodies and vesicles. Some may be lipid bodies while others may represent the site of synthesis and assembly of viral structural proteins. The large vacuoles appeared in cross section to be either empty, partially empty or full with electron-dense virus-like structures. Most of the virus-like particles had an electron-lucent core.

*Cytogenetics*

Cytogenetic studies on the original diagnostic marrow aspirate showed trisomy in the F group (19–20) in most cells. In some cells there was a 45-chromosome complement with absence of the Y chromosome and of a C or E18 chromosome. One cell with 44 chromosomes was seen, lacking a C8 and a D14 chromosome. G-banding was not sufficiently clear to distinguish whether the F-group trisomy involved Chromosomes 19 or 20 (Fig. 2).

Karyotype analysis after 9 months in culture revealed no normal spreads. Of the 33 spreads which were analysed, 32 were aneuploid (chromosome numbers 75–88) and one polyploid. All the aneuploid spreads contained one large submetacentric and a small acrocentric marker chromosome, while the polyploid spread contained 2 each of the large and small marker chromosomes. The increase in chromosome numbers could be seen in all groups (A–G) but it differed between the various spreads. This increase regularly included trisomy in the F group, similar to that seen in the original marrow cells, and also thought initially on grounds of size to be trisomy 19. However, satisfactory G-banding preparations revealed that the trisomy in fact involved Chromosome 20, which in this case appeared slightly longer than chromosome 19, but gave characteristic strong G-bands distally in both long and short arms (Fig. 3).

*Immunological markers*

Before culture, the patient's leucocytes were tested for EA(IgG), EA(IgM) and EAC receptors. 33% of his cells formed EA(IgG) rosettes and the other 2 markers were negative. After 9 months in culture the cells were virtually devoid of any of the immunological markers usually seen in cultured cells (Table II).

The entire cell population was found

FIG. 1.—Ultrastructure of a typical cell with lobulated nucleus. The cytoplasm contains several types of granules in addition to the numerous mitochondria. ×20,000.

FIG. 2.—Karyotype of the patient's marrow cells before culture, showing trisomy of Group F chromosome.

FIG. 3.—Karyotype of a cell after 9 months in culture, showing persistence of Group F trisomy. Giemsa banding showed that the trisomy is of Chromosome 20. In addition, there is an increase of chromosomes in all the other groups and the appearance of small (m) and large (M) marker chromosomes.

TABLE II.—*Immunological Marker Characteristics*

| | | % receptors | | | | |
|---|---|---|---|---|---|---|
| | Origin | E rosettes | Fc | C3 | SmIg | EBNA |
| Line 230* | Acute myelomonocytic leukaemia (PB) | 0 | 0 | 0 | 0 | 0 |
| Line K562 | Chronic myeloid leukaemia (PE) (Lozzio and Lozzio, 1975) | 5–9 | 90–95 | 3–9 | 0 | 0 |

* Marker analysis carried out on >300 cells in each case after 9 months of culture.
PB—peripheral blood; PE—pleural effusion.

to be EBNA− when tested after 6 and 9 months in culture.

### DISCUSSION

The malignant cells of most patients with acute myelogenous leukaemia are made up of EBNA− cells which do not have detectable surface receptors of the kind found on either B or T cells. In the past we have established and studied the properties of 18 cell lines from patients with acute myeloid leukaemia, and found that those cultures were EBNA+ (Karpas *et al.*, 1977*a*). Except for a single culture (Line 120) they also showed SmIg and had Fc and/or C3 receptors. Uncertainty remains as to whether these lines all represent proliferations of a sub-population of EBNA+ B cells, or whether in some cases a secondary *in vitro* infection of myeloid cells by EBV may have occurred, since some of the EBNA+ lines have been shown to possess myeloid characteristics (Karpas *et al.*, 1977*a*). However, morphologically they all appeared as undifferentiated blastoid cells.

In onr present communication we describe the properties of a cytologically and immunologically unique cell line which has been established from the peripheral blood of a patient with acute myelomonocytic leukaemia, and which appears to represent a proliferation of the malignant cells. Even after 9 months in culture they retain a myelomonoblastic morphology. After 6 months in culture about 50% of the cells still stained lightly with Sudan black, but after 9 months in culture Sudan black staining was lost. Weak scattered granular positivity to α-naphthyl butyrate esterase was, however, retained a feature more compatible with a monocytic lineage, than with B-cell lineage.

The cytogenetic studies also lend support to the malignant origin of the cells. Karyotype analysis of fresh patient's marrow cells before chemotherapy was started revealed trisomy of an F group chromosome. However, after prolonged culture the karyotype analysis revealed aneuploidy with variable extra chromosomes, but including the persistence of extra chromosomes in the F19–20 group, and the presence of large submetacentric and small abnormal acrocentric marker chromosomes. Very large submetacentric marker chromosomes have been described in fresh cells from a patient with acute leukaemia (Sandberg *et al.*, 1968). We have also found similar patterns of karyotype abnormalities in cultured murine cells transformed by avian and murine C-type viruses (Karpas *et al.*, 1971, 1972). Likewise, a report on the karyotype analysis of the human cell line which has been derived from a patient with chronic myeloid leukaemia revealed aneuploidy and polyploidy with abnormal marker chromosomes which appeared after prolonged *in vitro* culture, in spite of the fact that *in vivo* only the Ph chromosome abnormality could be detected (Lozzio and Lozzio, 1975). The ultrastructural examination of our culture at 6 months also revealed cells with myelomonoblastic morphology (Fig. 1). However, the most striking feature at this time was the membrane-bound vesicles which were sometimes packed with numerous electron-dense virus-like particles. The nature and properties of those particles is the subject of a separate report.

The immunological studies revealed that after 9 months in culture the cells were EBNA— and devoid of any known immunological markers, as were the majority of the fresh leukaemic cells. Thus, the immunological studies lend further support to the malignant origin of the cultured cells. The findings are compared in Table II with those in the only other established SmIg— EBNA— myeloid cell line, derived from chronic myeloid leukaemia (Lozzio and Lozzio, 1975).

The availability of this unique cell line, which represents an *in vitro* proliferation of leukaemic cells of apparently myeloid origin, may be of considerable value in further investigations of the block in differentiation manifest in acute leukaemia. These cells may also be useful in *in vitro* evaluation of new drugs to be used in the treatment of acute myeloid leukaemia (Holms and Little, 1974). Still more important, however, is the presence of membrane-bound vesicles containing virus-like particles. Evidence that these particles are indeed viral in nature, producing reverse transcriptase will be separately reported.

### ADDENDUM

The cells were also incubated with an antiserum against the Ia-like antigen complex in an indirect immunofluorescence test (Schlossman *et al.*, 1976, *Proc. natn. Acad. Sci. U.S.A.*, **73**, 1288). All the fresh patient leukaemic cells showed a very weak, but definite positivity, while the cultures cells were strongly positive.

We are grateful to Mr J. Emmines for help with the preparation of the electron micrographs. The work was supported by the Leukaemia Research Fund, U.K.

### REFERENCES

Burns, G. F., Cawley, J. C., Barker, C. R., Goldstone, A. H. & Hayhoe, F. G. J. (1977*a*) New Evidence Relating to the Nature and Origin of the Hairy Cells of Leukaemic Reticuloendotheliosis. *Br. J. Haemat.* **36**, 71.

Burns, G. F., Cawley, J. C., Flemans, R. J., Higgy, K. E., Worman, C. P., Barker, C. R., Roberts, B. E. & Hayhoe, F. G. J. (1977*b*) Surface Markers and Other Characteristics of Gaucher's Cells. *J. clin. Path.*, **30**, 981.

Cawley, J. C. & Hayhoe, F. G. J. (1973) *Ultrastructure of Haemic Cells.* Philadelphia: W. B. Saunders Co. p. 3.

Hayhoe, F. G. J. & Cawley, J. C. (1972) Acute Leukaemia: Cellular Morphology, Cytochemistry and Fine Structure. *Clinics in Haemat.*, **1**, 49.

Holms, H. L. & Little, J. M. (1974) Tissue Culture Microtest for Predicting the Response of Human Cancer Cells to Chemotherapy. *Lancet*, **i**, 985.

Karpas, A., Cawley, J., Tuckerman, E., Flemans, R. J. & Hayhoe, F. G. J. (1971) Cytochemistry, Cytogenetics and Ultrastructure of Hamster Tumour Cells Carrying Mouse Sarcoma Viral Genome (HT-1 Cells). *Br. J. Cancer*, **25**, 779.

Karpas, A., Cawley, J. & Tuckerman, E. (1972) Cytological, Cytogenetic and Ultrastructural Studies of Rat Cell Lines carrying Avian and Murine Sarcoma Genomes. *Z. Krebsforsch.*, **78**, 51.

Karpas, A., Hayhoe, F. G. J., Greenberger, J. S., Barker, C. R., Cawley, J. C., Lowenthal, R. M. & Moloney, W. C. (1977*a*) The Establishment and Cytological, Cytochemical and Immunological Characterisation of Human Haemic Cell Lines: Evidence for Heterogeneity. *Leukaemia Res.*, **1**, 35.

Karpas, A., Sandler, R. M. & Thorburn, R. J. (1977*b*) Null Cell Properties of a Lymphoid Cell Line from a Child with Acute Lymphoblastic Leukaemia. *Br. J. Cancer*, **36**, 177.

Ling, N. R., Bishop, S. & Jefferis, R. (1977) Use of Antibody-coated Red Cells for the Sensitive Detection of Antigen and in Rosette Tests for Cells Bearing Surface Immunoglobulins. *J. immunol. Meth.*, **15**, 279.

Lozzio, C. B. & Lozzio, B. B. (1975) Human Chronic Myelogenous Leukaemia Cell Line with Positive Philadelphia Chromosome. *Blood*, **45**, 321.

Reedman, B. M. & Klein, G. (1973) Cellular Localization of an Epstein–Barr (EBV) associated Complement-fixing Antigen in Producer and Non-producer Lymphoblastoid Cell Lines. *Int. J. Cancer*, **11**, 499.

Sandberg, A. A., Takagi, N., Sofuni, T. & Crosswhite, L. H. (1968) Chromosomes and Causation of Human Cancer and Leukaemia. V. Karyotypic Aspects of Acute Leukaemia. *Cancer, N.Y.*, **22**, 1268.